# Relationship between Mental Disorders and Optimism in a Community-Based Sample of Adults

**DOI:** 10.3390/bs12020052

**Published:** 2022-02-17

**Authors:** Ece Elif Öcal, Zeynep Demirtaş, Burcu Işıktekin Atalay, Muhammed Fatih Önsüz, Burhanettin Işıklı, Selma Metintaş, Çınar Yenilmez

**Affiliations:** 1 Ardahan Provincial Health Directorate, Ardahan 75000, Turkey; 2 Ünye District Health Directorate, Ünye 52300, Turkey; zeynpdemirtas@gmail.com; 3 Tepebaşı District Health Directorate, Tepebaşı 26120, Turkey; burcustkn@hotmail.com; 4 Department of Public Health, Faculty of Medicine, Eskişehir Osmangazi University, Eskişehir 26040, Turkey; monsuz@ogu.edu.tr (M.F.Ö.); burhanettinisikli@gmail.com (B.I.); selmamet@ogu.edu.tr (S.M.); 5 Department of Mental Health and Diseases, Faculty of Medicine, Eskişehir Osmangazi University, Eskişehir 26040, Turkey; cyenilmez@ogu.edu.tr

**Keywords:** mental disorders, PRIME MD, optimism, LOT, community

## Abstract

Optimism should be included in efforts to protect mental health, as it can provide cognitive resources. Optimism also reduces the negative effects of stressful life events associated with the occurrence and recurrence of mental disorders. This study aimed to evaluate the relationship between mental disorders and optimism in a community-based sample of adults. The study was conducted in three semi-rural clusters determined via random sampling. After adjustment in accordance with the independent variables, the relationship between each psychiatric disorder and Life Orientation Test (LOT) was calculated using logistic models. Overall, 24.5% of participants were categorized into at least one mental disorder group, with 20.8%, 3.5%, and 0.3% having one, two, or three mental disorders, respectively. The median LOT score was lower in patients diagnosed from the Primary Care Evaluation of Mental Disorders modules, except for the somatoform disorder module. Maintaining an optimistic view reduced the risk of mood disorders by 0.86 (OR; 95% CI, 0.81–0.91), anxiety disorders by 0.89 (0.83–0.97), and probable alcohol abuse by 0.83 (0.74–0.93) times after adjustment. The role of an optimistic view in coping with mental problems should be investigated in detail.

## 1. Introduction

Optimism is a way of thinking and involves expecting positive results from life. The direct effect of optimistic thinking is how people feel about themselves when they encounter problems. Optimistic people expect good results even in difficult situations. The effects of optimism go beyond just making an individual feel better and provide an important potential for what people can do when they face problems [1,2]. Many studies have associated optimism with physical disorders. These studies indicated that optimists exhibit more self-care and physical well-being and less pain in chronic illnesses than pessimists [3,4,5].

The relationship between optimism and mental disorders has been studied to a lower extent than that between optimism and physical disorders. Various studies have reported a positive effect of optimism on anxiety and depression [6,7]. At the most basic level, optimism is, by definition, inversely proportional with hopelessness, which is a risk factor for depressive disorders and acclaimed as a condition related to clinical psychology [8]. The effects of optimism were sequentially mediated by cognitive and behavioral variables [9].

Mental health is an integral and essential component of health. Many social, psychological, and biological factors determine a person’s mental health level [10]. Mental well-being is associated with mental and psychological well-being. The efforts adopted by the World Health Organization for improving the mental health of individuals and communities include the development of mental well-being, the prevention of mental disorders, the protection of human rights, and the care of individuals with mental disorders [11]. Optimism should be included in efforts to protect mental health, as it can provide cognitive resources. This is also related to the effect of optimism on reducing the negative effects of stressful life events associated with the occurrence and recurrence of mental disorders. Thus, optimism should be evaluated in efforts to improve the mental health of individuals and communities [8].

To guide health policies and studies regarding the protection of community mental health, information from community-based studies on an optimistic approach and mental health is required. The present study aimed to evaluate the relationship between mental disorders and optimism in a community-based sample of adults.

## 2. Materials and Methods

This cross-sectional study was conducted in 2017 in Eskisehir. The study was conducted in three semi-rural clusters (Sivrihisar, Beylikova, and Alpu) determined using random sampling. Ethical approval and administrative permissions were obtained for the study.

The study was conducted according to the guidelines of the Declaration of Helsinki, and approved by Ethics Committee of Non-Invasive Clinical Research of Eskişehir Osmangazi University (Approval Number: 09 and Date of Approval: 6 March 2017). Informed consent was obtained from all participants involved in the study.

The sample size was calculated to be at least 220 in each cluster to determine the decrease in the optimism scale score by 1 point with mental disorders with 95% confidence (α: 0.05) and 80% power (β: 0.20). The study group included 607 participants aged ≥18 years who applied to primary health care centers in the sampling areas for any reason and agreed to participate in the study. Individuals were informed regarding the purpose of the study, and then, their informed consent was obtained. Questionnaires were completed by the researchers using the face-to-face interview technique.

The first section of the questionnaire form, which was prepared by using the literature [12,13,14], had questions regarding the socio-demographic characteristics of the participants (age, sex, marital status, working status, income level, and education status) and backgrounds (presence of physician-diagnosed chronic illness). The second section included Primary Care Evaluation of Mental Disorders (PRIME MD)- and Life Orientation Test (LOT)-related questions.

PRIME MD was developed by Spitzer et al. in 1994 [15], and the Turkish validity and reliability study was conducted by Corapcioglu et al. in 1996 [16]. PRIME MD is a fully structured primary scale for screening the most common mental disorders, such as mood disorders, anxiety disorders, somatoform disorders, and probable alcohol abuse in primary health care centers. The scale comprises of two forms: a patient health questionnaire and a clinician evaluation guide. The patient health questionnaire is answered by the patient and consists of 26 questions with yes–no answers. A “yes” response to relevant sections of the patient health questionnaire translates into mood, anxiety, alcohol, or somatoform modules in the “clinician evaluation guide.”

The LOT was developed by Scheier et al. in 1987 [17], and its Turkish validity and reliability study was conducted by Aydin et al. in 1991 [18]. The LOT comprises 12 items: four reflect an optimistic view of life; four reflect a pessimistic view; and the remaining four serve as filler. Options range from 0 (strongly disagree) to 4 (strongly agree). The total score ranges between 0–32, and the higher the score, the more optimistic the view of life.

**Figure 1:**
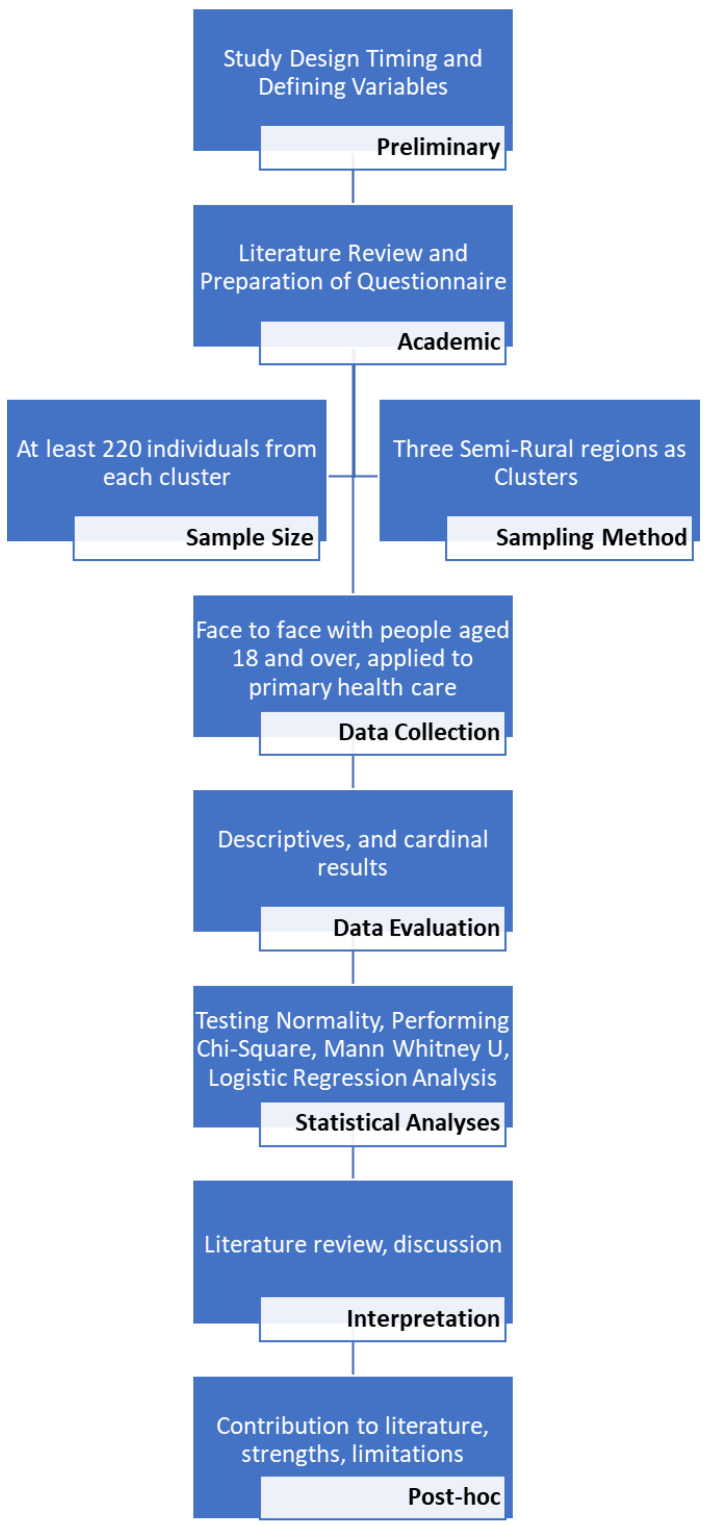
The study procedure.

The total LOT score was tested using the Kolmogorov–Smirnov normality test and graphs. The data obtained were evaluated using SPSS v. 15.0 (SPSS Inc., Chicago, IL, USA). A Mann–Whitney U test was used for statistical analysis, and a Chi-square (X^2^) test was used to compare the qualitative variables. The presence of psychiatric disorders was the dependent variable. Socio-demographic and background characteristics associated with psychiatric disorders were the independent variables, with a significance value set at *p* < 0.20. After adjustment in accordance with the independent variables (age, sex, education status, working status, income level, physician-diagnosed chronic illness background, and general health status), the relationship between each psychiatric disorder and LOT was calculated using logistic models. The most suitable model was selected. The study procedure is shown in Figure 1.

## 3. Results

The study group included 607 participants, of which 55.2% (n = 335) were males aged from 18–83 years with a mean (SD) of 43.37 (15.20) years. Overall, 47.8% (n = 290) of the participants had a primary school level education, 70.8% (n = 430) of them were married, 47.1% (n = 286) had a source of income, 34.6% (n = 210) had a physician-diagnosed chronic illnesses, and 42.6% (n = 259) declared their general health status as good.

**Figure 2:**
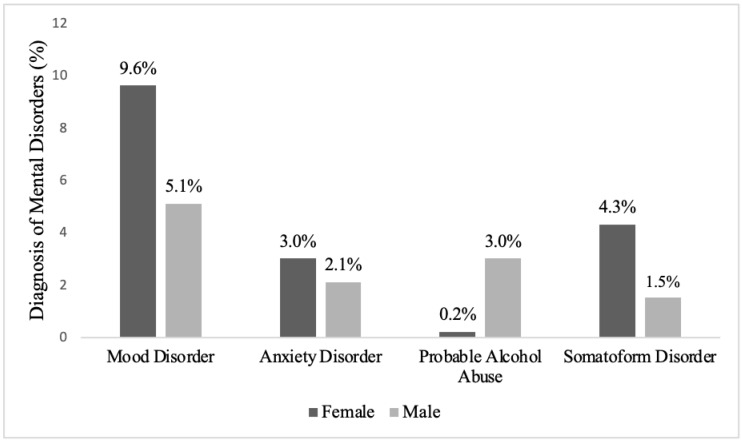
The distribution of mental disorders was obtained from the PRIME MD modules according to sex.

Of the study group, 24.5% (n = 149) were categorized into at least one mental disorder group, with 20.8% (n = 126), 3.5% (n = 21), and 0.3% (n = 2) being diagnosed with one, two, or three mental disorders, respectively. The distribution of mental disorders was obtained from the PRIME MD modules according to sex, as shown in Figure 2.

Mood disorders were more common among women and among those who were illiterate, did not have a job with regular income, declared poor income, had a physician-diagnosed chronic illness, or reported poor general health. Anxiety was more common among those aged from 25–44 and who had poor income and general health statuses. Probable alcohol abuse was higher among males. Somatoform disorders were more common among women and those who did not have jobs with regular income. The distribution of mental disorder diagnoses obtained from PRIME MD modules according to socio-demographic characteristics in the study group is shown in Table 1.

The LOT scores of participants ranked between 6 and 31, and the mean (SD) was 19.04 (4.61) with a median of 19.0. The median LOT score was lower in patients diagnosed from PRIME MD modules, except for the somatoform disorder module. The comparison of LOT scores in the study group based on the mental disorder diagnoses obtained from PRIME MD is shown in Table 2.

After adjusting for age, sex, education status, marital status, working status, income level, physician-diagnosed chronic illness background, and general health status in a multi-logistic regression analysis, it was observed that with an optimistic view, the risk of mood disorders decreased by 0.86 (OR; 95% CI, 0.81–0.91) times, of anxiety disorder decreased by 0.89 (0.83–0.97) times, and of probable alcohol abuse decreased by 0.83 (0.74–0.93) times. Table 3 shows the results of the multi-logistic regression model performed to determine the relationship between the mental disorder diagnoses obtained from PRIME MD and LOT scores, after adjusting for related factors in the study group.

## 4. Discussion

This study demonstrated the effect of an optimistic view on mental health in a community-based sample. In the literature, there are limited numbers of studies investigating the impact of optimism on mental health. The study group comprised adults aged ≥18 years. The three clusters included in the study of locations, cultures, industries, etc., were very similar. Mood disorders were the most common mental disorders in this group. Similarly, many studies conducted using data from PRIME MD also confirmed that mood disorders were the most common mental disorders [12,13,14,19,20].

In this study, mood disorders were the most common mental disorders and were influenced by three fundamental socio-demographic elements. One of these elements was female sex. Several studies conducted using data from PRIME MD have demonstrated that mood disorders are more common among woman [12,13,19,20]. In general, mental disorders are more common among women [21]. The more frequent presence of mood disorders in women may be the result of biological, social, cultural, and sociological factors. In this respect, the effect of neuroendocrine factors on sex and the male dominance of society is important. As a result, women may have a more traumatic life. In addition, women may undertake family responsibility more intensively, especially in childcare, which may affect the result. A young age was found to be the second factor affecting mood disorders. A study conducted by Bilge et al. in the same city demonstrated that in younger age groups, the rate of diagnosis from PRIME MD was higher [12]. In the study conducted by Grandes et al. with a different scale, age was an effective factor. Although mood disorders were high among people in 40s, those in their 30s had the highest rate of anxiety disorders [22]. Reportedly, mental disorders constitute a large part of the illness burden in young people in all societies [23]. Although social stressors are more influential in younger age groups, the elderly are more resilient against social stressors, which may explain this consequence [6,19]. Poor general health was the last factor affecting mood disorders. People who define their general health as poor are more likely to have comorbid disorders, especially chronic diseases. In addition, the quality of life of these people is affected adversely. In this respect, the presence of chronic or comorbid diseases may increase mood disorders. There are studies supporting this rationale in the literature [20,22]. Patients diagnosed with chronic diseases need to reorganize their lives according to their illness and treatment programs. Therefore, this could lead to mood disorders such as depression, by influencing their illness independence, lifestyle, and other personal perceptions. Moreover, physical, mental, and social changes that may develop due to chronic diseases may trigger mental disorders, and people may react psychologically to the difficulties associated with disease [24]. Therefore, early diagnosis and treatment of various mental disorders of such patients is important in primary care. Physicians should evaluate and help patients with poor general health in terms of mental disorders.

Our study could not determine the relationship between optimism level and somatoform disorders. Reportedly, optimism is a strongly associated with reduced depression, anxiety, and somatization [25]. In addition, a negative correlation between optimism and somatization has been reported in several studies [25,26]. In the presence of any stressor or emotion, somatization manifests as physical reactions rather than emotional or cognitive reactions. Mental problems arise with physical symptoms [27]. Somatoform disorders have been associated with a high number of comorbidities, including anxiety or depressive disorders [28,29]. The fact that somatoform disorders manifest themselves with physical symptoms and make people think about presence of physical reasons may lead them not to be associated with optimism. Moreover, the association of somatization with other mental disorders such as anxiety and/or depression may be considered a confounding and obscuring factor.

The assessment of only optimism on mental disorders revealed that people with mood disorders, anxiety disorders, and probable alcohol abuse had a lower level of optimism. A negative correlation was noted between optimism and the depression, anxiety, pain weakness, and post-traumatic diagnosis scale in a community-based study [6]. In addition, a study conducted among university students demonstrated a negative correlation between optimism and anxiety, depression, and emotional stress [26]. In this context, it is normal that optimism positively affects an individual’s physical health besides her/his mental health, and the results of the study confirm this.

Based on the results of the present study, factors that might have had an association with mental disorders were adjusted to assess the impact of optimism on mental disorders. Following adjustment, an optimistic view of life was found to decrease the risk of mood disorders, anxiety disorders, and probable alcohol abuse. In the literature, there are studies indicating a negative correlation between optimism and mood disorders, especially depression [8,30]. Another study stated that pessimists had very little hope for the future when compared with optimists, and they had a higher risk of depressive and anxiety disorders owing to impaired social functioning and quality of life [31]. Optimism is important in understanding the fragility of mental disorders, especially mood disorders. High optimism is related with lower stress levels, increased social support, and outstanding coping strategies, which are protective against depression [32]. While trying to cope with the difficulties they encounter, individuals with an optimistic view distance themselves from pessimism and hopelessness, which ensures good mental health. This situation makes us consider that some mental disorders may be decreased by changing one’s point of view.

Studies have demonstrated a negative relationship between optimism and anxiety disorders, similar to mood disorders [30,33]. Optimism has been consistently associated with positive outcomes such as subjective well-being and physical health. Moreover, optimism motivates active and persistent coping behaviors that are beneficial during stressful situations [34]. Optimism is a mental attitude that can cope with the pressures of daily social and working life, in addition to its significant effect on physical and mental health [35]. While confronting problems, individuals show a wide range of emotions ranging from excitement and willingness to anger to anxiety and depression. A balance of these emotions is related to the differences in optimism. While optimists think of good results and provide a positive mix of emotions even under difficult situations, pessimists expect bad results. This brings negative feelings such as anxiety, anger, sadness, and even despair. In this context, optimism and pessimism are related to the emotions that people feel when they encounter problems, and they show their effect clearly when individuals cope with difficulties [8].

Reportedly, optimism is associated with moderate alcohol drinking. This also supports the results of our study that an optimistic view of life decreases the risk of probable alcohol abuse [36,37]. The results of our study are similar to those reported previously. Optimism is protective against mental and physical health problems and related to good social health consequences [8]. In addition, optimism and pessimism are related to the fundamental components of mental situation and personality. While pessimism is associated with negative emotions and neuroticism, optimism is related to primarily extroverted positive emotions [38]. Optimism provides individuals with the opportunity to quickly and efficiently determine their expectations for coping with several challenges and for overcoming problems with a solution-oriented approach.

Our study has some limitations. The first is related to the utilization of PRIME MD. Reportedly, PRIME MD has a sensitivity of 83%, a specificity of 88%, and a positive predictive value of 80%, which puts subthreshold symptoms into the disease category and thus might have led to a high prevalence of mental disorders [19,39]. In addition, PRIME MD can diagnose only four groups of illnesses: mood, alcohol, anxiety, and somatoform modules; other mental illnesses cannot be categorized, which is another limitation of the scale. The second limitation is that the study was conducted by voluntary participants. In this regard, people who had mental illnesses, whose relatives had mental illnesses, or people who were interested in the subject might have participated the study. Another dimension of the subject is the possibility of people not answering the questions honestly because of the fear of stigmatization. The third limitation is that the study was cross-sectional, preventing any causal outcome.

However, our study also has strengths. Firstly, our study was conducted in a semi-rural area, via a community-based sampling method, and at a primary health care center. Secondly, there are a limited number of studies investigating the relationship between optimism and mental illnesses among people visiting primary health care centers. Therefore, our study will present an important contribution to the literature. Finally, although we have mentioned certain limitations of the PRIME MD, the utilization of this scale is a strength of our study. This is because PRIME MD is a standard diagnostic tool designed for primary care, which is easy to apply and has a short application time [15].

## 5. Conclusions

This study contributes to the evidence that an optimistic approach protects and promotes mental health. According to PRIME MD, individuals who were diagnosed with mood disorders, anxiety disorders, and probable alcohol abuse had lower optimism scores, while those with somatoform disorders were not affected. In another study conducted with PRIME MD in the city center of the same city as our study, it was reported that the most common mental disorders were mood disorders [13]. This indicates that similar results can occur in both urban and semi-rural populations.

Primary health care centers, where we conducted our study, are the first places that mental illnesses are noticed and diagnosed. Therefore, it is necessary to evaluate people who visit primary health care centers for their mental health. An optimistic point of view, which can be mentioned among personal characteristics, positively contributes to an individual’s mental condition. Implementing educational activities to gain a more optimistic and positive attitude toward life can contribute to the mental health of people. In the future, more complicated studies evaluating mental health and optimism together should be conducted. In these studies, the role of an optimistic point of view for coping with mental problems should be investigated and evaluated in detail by suggesting other variables.

## Figures and Tables

**Table 1:** The distribution of mental disorder diagnoses obtained from PRIME MD modules according to the socio-demographic characteristics in the study group.

		Mood Disorder	Anxiety Disorder	Probable Alcohol Abuse	Somatoform Disorder
		Yes (%) *	Yes (%) *	Yes (%) *	Yes (%) *
Age Group (year)	24 and below	11.3	2.8	4.2	2.8
	25–44	16.9	8.5 **	4.2	6.5
	45–64	13.5	2.9	1.4	6.2
	65 and above	13.2	1.5	2.9	4.4
*p*		0.559	0.013	0.350	0.628
Sex	Male	9.3	3.9	5.4	2.7
	Female	21.3	6.6	0.4	9.6
*p*		**<0.001**	0.181	**0.001**	**0.001**
Education Status	Illiterate	29.8 **	8.8	0.0	10.5
	Primary School	15.9	4.5	4.1	6.6
	High school	9.0	5.5	3.4	2.8
	University and above	11.3	4.3	1.7	5.2
*p*		**0.001**	0.572	0.309	0.159
Marital status	Married	13.3	5.1	2.8	5.6
	Single	18.1	5.0	4.0	6.2
*p*		0.127	1.000	0.623	0.910
Working status	Yes	9.8	4.2	2.1	3.1
	No	19.0	5.9	4.0	8.1
*p*		**0.001**	0.437	0.252	**0.015**
Income level	Good	11.2	3.4	0.0 **	2.6
	Medium	12.8	4.4	3.4	6.6
	Poor	28.6 **	10.7 **	6.0	6.0
*p*		**<0.001**	**0.039**	**0.023**	0.256
Physician-diagnosed chronic illness background	No	12.6	4.0	3.5	4.8
	Yes	18.6	7.1	2.4	7.6
*p*		**0.048**	0.143	0.559	0.214
General Health Status	Good	7.3	3.4	2.7	4.6
	Poor	29.9	8.6	4.1	8.1
*p*		**<0.001**	**0.011**	0.507	0.124

* Row percentage is given. ** Items that provided a difference between advanced X2 test are marked.

**Table 2:** The comparison of LOT scores in the study group based on the mental disorder diagnoses obtained from PRIME MD.

Mental Disorder Group		Life Orientation Test Score		*p*
		Mean ± SD	Median (Min.–Max.)	
Mood Disorder	Yes	15.67 ± 4.04	15.0 (6.0–26.0)	
	No	19.61 ± 4.45	20.0 (7.0–31.0)	**<0.001**
Anxiety Disorder	Yes	16.74 ± 3.12	17.0 (11.0–23.0)	
	No	19.16 ± 4.65	19.0 (6.0–31.0)	**0.001**
Probable Alcohol Abuse	Yes	15.11 ± 3.81	15.0 (9.0–24.0)	
	No	19.16 ± 4.58	19.0 (6.0–31.0)	**<0.001**
Somatoform Disorder	Yes	18.23 ± 4.06	19.0 (9.0–26.0)	
	No	19.09 ± 4.64	19.0 (6.0–31.0)	0.266

**Table 3:** The results of the multi-logistic regression model performed for determining the relationship between the mental disorder diagnoses obtained from PRIME MD and LOT scores, after adjusting for related factors in the study group.

	Mood Disorder OR (%95 CI)	Anxiety Disorder OR (%95 CI)	Probable Alcohol Abuse OR (%95 CI)	Somatoform Disorder OR (%95 CI)
Age	0.63 (0.44–0.91) *	0.45 (0.27–0.75) **	0.71 (0.41–1.24)	0.99 (0.61–1.61)
Gender	2.59 (1.47–4.53) **	1.60 (0.75–3.40)	0.06 (0.008–0.44) **	3.08 (1.35–7.02) **
Education status	1.31 (0.90–1.90)			1.03 (0.63–1.70)
Marital status	0.78 (0.45–1.37)			
Working status	0.84 (0.44–1.60)			1.64 (0.65–4.13)
Income level	1.12 (0.67–1.85)	1.71 (0.86–3.42)	2.08 (0.87–5.02)	
Physician-diagnosed chronic illness background	1.34 (0.75–2.41)	2.60 (1.12–6.05) *		
General health status	3.81 (2.19–6.64) ***	2.09 (0.92–4.73)		1.43 (0.67–3.04)
Total score of Life Orientation Test	0.86 (0.81–0.91) ***	0.89 (0.83–0.97) **	0.83 (0.74–0.93) **	0.98 (0.91–1.06)

* <0.05; ** <0.01; *** <0.001; OR: Odds ratio; CI: Confidence interval.

## Data Availability

The data presented in this study are available on reasonable request from the corresponding author.

## References

[1] Scheier M.F., Carver C.S. (1992). Effects of optimism on psychological and physical well-being: Theoretical overview and empirical update. Cogn. Ther. Res..

[2] Carver C.S., Scheier M.F. (1998). Discrepancy-Reducing Feedback Processes in Behavior. On the Self-Regulation of Behavior.

[3] De Ridder D., Fournier M., Bensing J. (2004). Does optimism affect symptom report in chronic disease? What are its consequences for self-care behaviour and physical functioning?. J. Psychosom. Res..

[4] Fournier M., de Ridder D., Bensing J. (2002). Optimism and adaptation to chronic disease: The role of optimism in relation to self-care options of type 1 diabetes mellitus, rheumatoid arthritis and multiple sclerosis. Br. J. Health Psychol..

[5] Affleck G., Tennen H., Zautra A., Urrows S., Abeles M., Karoly P. (2001). Women’s pursuit of personal goals in daily life with fibromyalgia: A value-expectancy analysis. J. Consult. Clin. Psychol..

[6] Glaesmer H., Rief W., Martin A., Mewes R., Brähler E., Zenger M., Hinz A. (2012). Psychometric properties and population-based norms of the Life Orientation Test Revised (LOT-R). Br. J. Health Psychol..

[7] Myhren H., Ekeberg Ø., Tøien K., Karlsson S., Stokland O. (2010). Posttraumatic stress, anxiety and depression symptoms in patients during the first year post intensive care unit discharge. Crit. Care.

[8] Carver C.S., Scheier M.F., Segerstrom S.C. (2010). Optimism. Clin. Psychol. Rev..

[9] Ramsay J.E., Yang F., Pang J.S., Lai C.M., Ho R.C., Mak K.K. (2015). Divergent pathways to influence: Cognition and behavior differentially mediate the effects of optimism on physical and mental quality of life in Chinese university students. J. Health Psychol..

[10] World Health Organization (2018). Mental Health: Strengthening Our Response.

[11] World Health Organization (2019). Mental Health.

[12] Bilge U., Ünlüoğlu İ., Yenilmez Ç. (2012). Bir üniversite hastanesi dahiliye polikliniğine başvuran kronik bedensel hastalığı olan hastalarda ruhsal bozuklukların belirlenmesi [Determination of psychiatric disorders among outpatients admitted to the internal medicine clinic in a university hospital]. J. Neurol. Sci..

[13] Keskin A., Ünlüoğlu İ., Bilge U., Yenilmez Ç. (2013). The prevalence of psychiatric disorders distribution of subjects gender and its relationship with psychiatric help-seeking. Noro Psikiyatr. Arsivi.

[14] Keskin A., Bilge U. (2014). Mental disorders frequency alternative and complementary medicine usage among patients with hypertension and type 2 diabetes mellitus. Niger. J. Clin. Pract..

[15] Spitzer R.L., Williams J.B.W., Kroenke K., Linzer M., deGruy III F.V., Hahn S.R., Brody D., Johnson J.G. (1994). Utility of a new procedure for diagnosing mental disorders in primary care: The PRIME-MD 1000 study. JAMA.

[16] Çorapçıoğlu A., Köroğlu E., Ceyhun B., Doğan O. (1996). Birinci basamak sağlık hizmetlerinde psikiyatrik tanı koydurucu bir ölçeğin (Prime-MD) Türkiye için uyarlanması. Nöropsikiyatri Gündemi.

[17] Scheier M.F., Carver C.S. (1987). Dispositional optimism and physical well-being: The influence of generalized outcome expectancies on health. J. Personal..

[18] Aydın G., Tezer E. (1991). İyimserlik, sağlık sorunları ve akademik başarı ilişkisi. Psikol. Derg..

[19] Ansseau M., Dierick M., Buntinkx F., Cnockaert P., De Smedt J., Van Den Haute M., Vander Mijnsbrugge D. (2004). High prevalence of mental disorders in primary care. J. Affect. Disord..

[20] Canakci M.E., Acar N., Yenilmez C., Ozakin E., Kaya F.B., Arslan E., Caglayan T., Dolgun H. (2019). Frequency of Psychiatric Disorders in Nonemergent Admissions to Emergency Department. Niger. J. Clin. Pract..

[21] Van de Velde S., Boyd A., Villagut G., Alonso J., Bruffaerts R., De Graaf R., Florescu S., Haro J., Kovess-Masfety V. (2018). Gender differences in common mental disorders: A comparison of social risk factors across four European welfare regimes. Eur. J. Public Health.

[22] Grandes G., Montoya I., Arietaleanizbeaskoa M.S., Arce V., Sanchez A. (2011). The burden of mental disorders in primary care. Eur. Psychiatry.

[23] Patel V., Flisher A.J., Hetrick S., McGorry P. (2007). Mental health of young people: A global public-health Challenge. Lancet.

[24] Drozd W., Wojnar M., Araszkiewicz A., Nawacka-Pawlaczyk D., Urbanski R., Cwiklinska-Jurkowska M., Rybakowski J. (2007). The prevalance of depressive disorders in primary care in Poland. Wiad Lek.

[25] Grant H., Higgins E.T. (2003). Optimism, promotion pride, and prevention pride as predictors of quality of life. Pers. Soc. Psychol. Bull..

[26] Gustems-Carnicer J., Calderón C., Santacana M.F. (2017). Psychometric properties of the Life Orientation Test (LOT-R) and its relationship with psychological well-being and academic progress in college students. Rev. Latinoam. Psicol..

[27] Kesebir S. (2004). Depresyon ve somatizasyon. Klin. Psikiyatr..

[28] De Waal M.W.M., Arnold I.A., Eekhof J.A., Van Hemert A.M. (2004). Somatoform disorders in general practice: Prevalence, functional impairment and comorbidity with anxiety and depressive disorders. Br. J. Psychiatry.

[29] World Health Organization (2001). The World Health Report 2001: Mental Health: New Understanding, New Hope.

[30] Zenger M., Brix C., Borowski J., Stolzenburg J.U., Hinz A. (2010). The impact of optimism on anxiety, depression and quality of life in urogenital cancer patients. Psychooncology.

[31] Van der Velden P.G., Kleber R.J., Fournier M., Grievink L., Drogendijk A., Gersons B.P. (2007). The association between dispositional optimism and mental health problems among disaster victims and a comparison group: A prospective study. J. Affect. Disord..

[32] Szalma J.L. (2009). Individual differences in performance, workload and stress in sustained attention: Optimism and pessimism. Personal. Individ. Differ..

[33] Dolcos S., Hu Y., Iordan A.D., Moore M., Dolcos F. (2016). Optimism and the brain: Trait optimism mediates the protective role of the orbitofrontal cortex gray matter volume against anxiety. Soc. Cogn. Affect. Neurosci..

[34] Nes L.S., Segerstrom S.C. (2006). Dispositional optimism and coping: A meta-analytic review. Personal. Soc. Psychol. Rev..

[35] Conversano C., Rotondo A., Lensi E., Della Vista O., Arpone F., Reda M.A. (2010). Optimism and its impact on mental and physical well-being. Clin. Pract. Epidemiol. Ment. Health CP EMH.

[36] Giltay E.J., Geleijnse J.M., Zitman F.G., Buijsse B., Kromhout D. (2007). Lifestyle and dietary correlates of dispositional optimism in men: The Zutphen Elderly Study. J. Psychosom. Res..

[37] Anthony E.G., Kritz-Silverstein D., Barrett-Connor E. (2016). Optimism and Mortality in Older Men and Women: The Rancho Bernardo Study. J. Aging Res..

[38] Marshall G.N., Wortman C.B., Kusulas J.W., Hervig L.K., Vickers R.R. (1992). Distinguishing optimism from pessimism: Relations to fundamental dimensions of mood and personality. J. Personal. Soc. Psychol..

[39] Linzer M., Spitzer R., Kroenke K., Williams J.B., Hahn S., Brody D., DeGruy F. (1996). Gender, quality of life, and mental disorders in primary care: Results from the PRIME-MD 1000 study. Am. J. Med..

